# Fungi-Kcr: a language model for predicting lysine crotonylation in pathogenic fungal proteins

**DOI:** 10.3389/fcimb.2025.1615443

**Published:** 2025-07-15

**Authors:** Yong-Zi Chen, Xiaofeng Wang, Zhuo-Zhi Wang, Haixin Li

**Affiliations:** ^1^ Cancer Biobank, Tianjin Medical University Cancer Institute & Hospital, National Clinical Research Center for Cancer, Tianjin, China; ^2^ Key Laboratory of Molecular Cancer Epidemiology, Tianjin’s Clinical Research Center for Cancer, Tianjin, China; ^3^ College of Mathematics and Computer Sciences, Shanxi Normal University, Taiyuan, China; ^4^ School of Biomedical Engineering, Tianjin Medical University, Tianjin, China

**Keywords:** lysine crotonylation, fungal proteins, language model, pathogen, post-translational modifications

## Abstract

**Introduction:**

Lysine crotonylation (Kcr) is an important post-translational modification (PTM) of proteins, playing a key role in regulating various biological processes in pathogenic fungi. However, the experimental identification of Kcr sites remains challenging due to the high cost and time-consuming nature of mass spectrometry-based techniques.

**Methods:**

To address this limitation, we developed Fungi-Kcr, a deep learning-based model designed to predict Kcr modification sites in fungal proteins. The model integrates convolutional neural networks (CNN), gated recurrent units (GRU), and word embedding to effectively capture both local and long-range sequence dependencies.

**Results:**

Comprehensive evaluations, including ten-fold cross-validation and independent testing, demonstrate that Fungi-Kcr achieves superior predictive performance compared to conventional machine learning models. Moreover, our results indicate that a general predictive model performs better than species-specific models.

**Discussion:**

The proposed model provides a valuable computational tool for the large-scale identification of Kcr sites, contributing to a deeper understanding of fungal pathogenesis and potential therapeutic targets. The source code and dataset for Fungi-Kcr are available at https://github.com/zayra77/Fungi-Kcr.

## Introduction

Protein post-translational modifications (PTMs) play a pivotal role in regulating protein function, localization, and interactions, thereby influencing various cellular processes. Among the diverse array of PTMs, lysine crotonylation (Kcr) has emerged as a critical modification involved in gene regulation, metabolism, and cellular signaling. It was initially discovered in histones (Tan et al., 2011) and then subsequently identified in non-histone proteins (Wei et al., 2017; Xu et al., 2017; Wu et al., 2019; Hou et al., 2021), highlighting its widespread biological significance. For instance, in the small intestine crypt and colon, increased crotonylation at the lysine 18 site on histone H3 abundance at transcription start sites was associated with higher gene expression levels (Fellows et al., 2018). Crotonylation levels have also been reported to be changed in various cancers (Wan et al., 2019; Hou et al., 2024; Yang et al., 2024; Ji et al., 2025; Lin et al., 2025; Wang et al., 2025).

In fungi, particularly pathogenic species, crotonylation has emerged as a critical regulatory mechanism, potentially influencing fungal development, pathogenicity, and environmental adaptation. Targeting crotonylation sites holds significant promise for developing therapeutic interventions to combat fungal diseases in both humans and plants. For example, studies have revealed that crotonylation is more prevalent than acetylation and succinylation in Candida albicans, underscoring its biological importance. The functional significance of crotonylation appears to be closely associated with ribosomal biogenesis/protein translation, proteome regulation, and carbon metabolism/mitochondrial energy production (Xu et al., 2017) (Zhou et al., 2016; Zhou et al., 2021). In Trichophyton rubrum, a model organism for dermatophytes and anthropophilic pathogenic filamentous fungi, crotonylated proteins have been shown to participate in distinct pathways during the conidial and mycelial stages. These findings provide novel insights that could guide the development of antifungal agents (Xu et al., 2022). Similarly, Botrytis cinerea, a globally significant fungal pathogen responsible for gray mold disease in a wide range of hosts, presents an opportunity for further exploration. Investigating the functions of crotonylation in substrate proteins could deepen our understanding of the molecular mechanisms underlying B. cinerea pathogenesis at the protein level (Zhang et al., 2020).

Experimental methods for detecting lysine crotonylation sites employ a range of techniques designed to identify and quantify this post-translational modification in proteins. These include high-performance liquid chromatography (HPLC) fractionation, stable isotope labeling of amino acids in cell culture (SILAC), immunological affinity enrichment, high-resolution liquid chromatography-tandem mass spectrometry (LC-MS/MS), immunofluorescence microscopy, and chemical labeling and enrichment strategies (Jiang et al., 2024). While these biological experiments are the gold standard for Kcr site identification, they are often time-consuming, labor-intensive, and costly. Moreover, despite their high sensitivity, mass spectrometry-based platforms can only detect a subset of crotonylated peptides due to challenges such as protein abundance, incomplete protein hydrolysis, and variable digestion efficiency. These limitations underscore the need for complementary approaches to enhance Kcr site identification.

Initial models for predicting lysine crotonylation sites were limited by small, histone-specific datasets and relied on conventional machine learning methods like SVMs and random forests (Ju and He, 2017; Qiu et al., 2018; Malebary et al., 2019; Liu et al., 2020). While they were limited in scope and predictive power due to the restricted datasets, they laid the groundwork for subsequent advancements. Advances in mass spectrometry and deep learning have enabled the development of more robust models, such as Deep-Kcr (Lv et al., 2021), DeepCap-Kcr (Khanal et al., 2022), and BERT-Kcr (Qiao et al., 2022), which predict Kcr sites on both histones and non-histones, achieving significant performance improvements. Others are tailored to predict Kcr sites on non-histones, such as nhKcr (Chen et al., 2021), iKcr_CNN (Dou et al., 2022), and CapsNh-Kcr (Khanal et al., 2023). The primary input features of these models consist of binary encoding and embedding vectors.

While some models, like PlantNh-Kcr (Jiang et al., 2024), extend predictions for Kcr sites on non-histones in plants, there remains a critical gap in computational tools for fungi. Recent studies have identified Kcr sites in fungal species, emphasizing their biological importance in fungal physiology and pathogenicity. However, no dedicated models exist for fungal Kcr prediction, highlighting the need to integrate fungal Kcr data and develop specialized computational tools. Addressing this gap will advance our understanding of fungal biology and provide valuable insights into fungal-host interactions and potential therapeutic targets.

To address these challenges, we present Fungi_Kcr, a novel deep learning model specifically designed for the prediction of crotonylation sites in fungi. Built on advanced architectures, Fungi_Kcr integrates ​word embeddings to capture sequence-based features to accurately identify crotonylation sites across diverse fungal species. By leveraging large-scale fungal proteomic data, Fungi_Kcr overcomes the limitations of existing models and provides a robust, generalizable framework for PTM prediction in fungi. The development of Fungi_Kcr represents a significant step forward in the study of fungal crotonylation, offering new insights into the functional roles of this modification in fungal biology. Moreover, the model’s ability to predict crotonylation sites with high accuracy has practical implications for understanding fungal pathogenicity, identifying potential therapeutic targets, and advancing fungal biotechnology. This work underscores the potential of ​word embeddings and language models in bridging the gap between computational biology and experimental research, paving the way for future discoveries in the field of PTM biology.

## Materials and methods

### Benchmark dataset

We first collected lysine crotonylation sites from three fungal species, including 3,923 from Botrytis cinerea (Xu et al., 2017), 5,200 from Candida albicans (Zhou et al., 2021), and 12,543 from Trichophyton rubrum (Xu et al., 2022). Next, we retrieved the corresponding protein sequences from the UniProt database (Dimmer et al., 2012) for each species. From these sequences, we extracted peptides ranging in length from 23 to 33 residues. Based on performance evaluation, a window size of 31 residues yielded the highest AUC (**Supplementary Figure 1**). In this setting, the central lysine (K) residue was positioned at the 16th position, flanked by 15 upstream and 15 downstream residues. If a peptide lacked sufficient residues on one side, we padded the missing positions with “X.” Peptides where the central K residue corresponded to an experimentally verified Kcr site were labeled as positive samples, while peptides with a central unmodified lysine were labeled as negative samples. This resulted in 30,213 negative peptides from Botrytis cinerea, 70,639 from Trichophyton rubrum, and 59,793 from Candida albicans.

**Figure 1:**
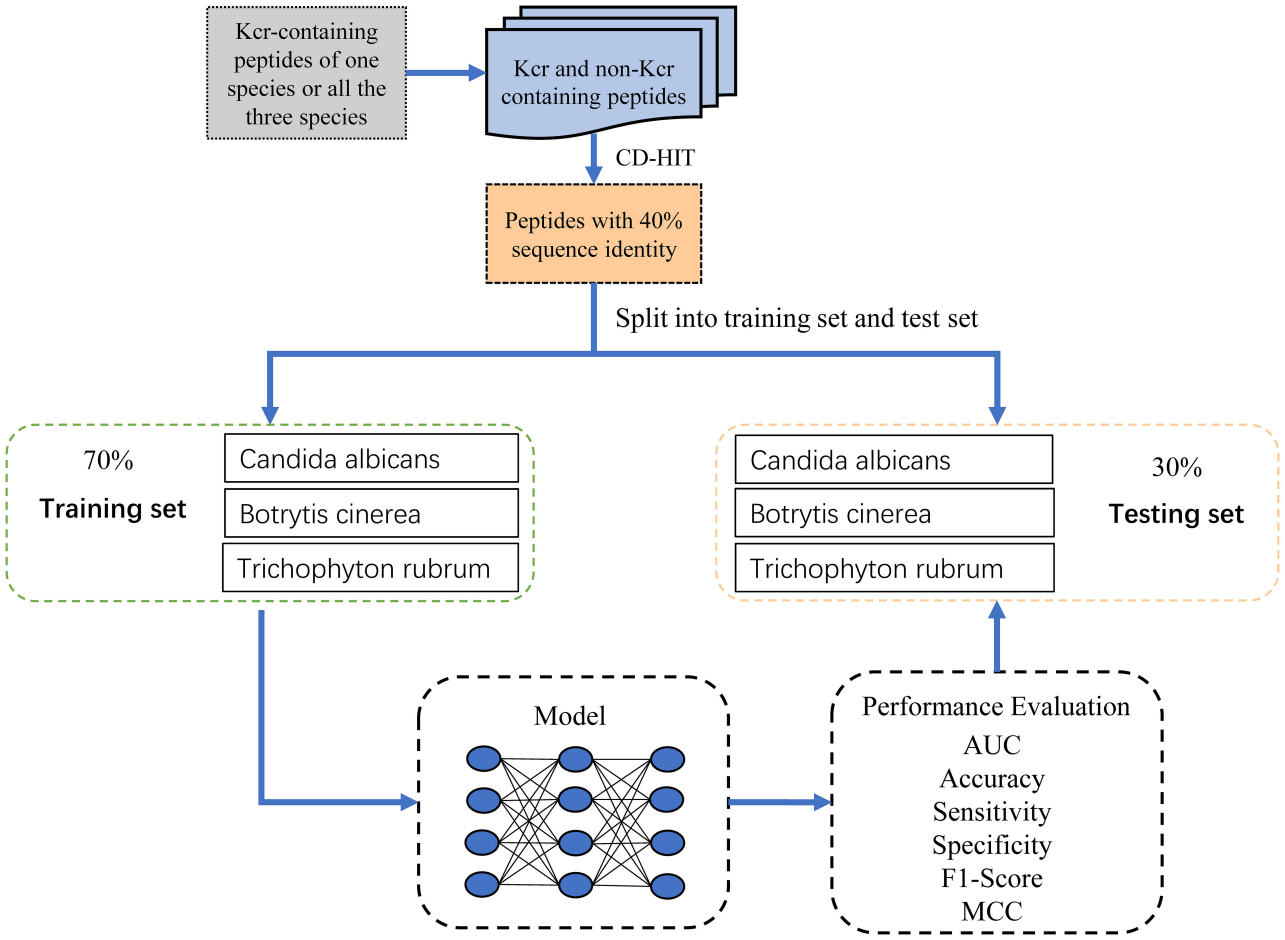
Flowchart illustrating the dataset preparation process.

To reduce redundancy and minimize potential false negatives, we applied the CD-HIT program (Huang et al., 2010) with a sequence identity threshold of 40%, resulting in 9,626 non-redundant positive samples. An equal number of negative samples (9,626) were randomly selected to ensure a balanced dataset for model training. To assess model performance across species, we stratified the samples accordingly. For each species, we randomly partitioned the samples into training and test sets at a 7:3 ratio while maintaining the balance between positive and negative samples. The training dataset comprised 1,780, 2,261, and 4,969 samples for Botrytis cinerea, Candida albicans, and Trichophyton rubrum, respectively, totaling 6,738 samples, with a 1:1 positive-to-negative ratio. Similarly, the test dataset contained 763, 970, and 2,130 samples for the respective species, totaling 2,888 samples, also maintaining a 1:1 ratio. The sample collection process was meticulously designed, as illustrated in **Figure 1**, and the detailed distribution of samples across the training and test datasets is provided in **Table 1**.

**Table 1:** The number of positive and negative samples for each species in the training and testing datasets.

Species	Training set		Testing set	
	Positive	Negative	Positive	Negative
Botrytis cinerea	1,780	1,780	763	763
Candida albicans	2,261	2,261	970	970
Trichophyton rubrum	4,969	4,969	2,130	2,130
Combined dataset	6,738	6,738	2,888	2,888

### Encoding methods

To transform amino acid sequence samples in our dataset into numerical representations suitable for model input, we employed a method that combines word embedding with positional encoding. Word embedding is a widely used technique in both natural language processing (NLP) and bioinformatics, mapping words into a low-dimensional vector space (Nguyen et al., 2019; Pratyush et al., 2023; Mahmud et al., 2024; Asim et al., 2025). This approach ensures that semantically similar words are represented by vectors that are close to each other in this space. In our context, peptides are treated as sentences, with each amino acid residue acting as a word. Initially, the 20 standard amino acid residues are assigned unique integer values ranging from 0 to 19. These integers are then transformed into low-dimensional vectors, denoted as *d_model*, allowing the model to capture semantic relationships between amino acids. While word embedding effectively encodes semantic similarity, it lacks the ability to represent the sequential order of amino acids in protein sequences. To address this limitation, we incorporated positional encoding (Yuan et al., 2023; Shi et al., 2025). Positional encoding is computed using sine and cosine functions, with the formula given as follows:


$$PE\left(pos,2i\right)=sin\left(\frac{pos}{10000^{2i/d_{model}}}\right)$$

$$PE\left(pos,2i+1\right)=cos\left(\frac{pos}{10000^{2i+1/d_{model}}}\right)\;$$

Here, *pos* represents the position of an amino acid in the sequence, while *2i* and *2i+1* index the components of the positional encoding vector, which has the same dimensionality as *d_model*. We adopted sinusoidal functions—sine for even dimensions and cosine for odd dimensions—because they encode unique position information across multiple frequencies, enabling the model to learn both absolute and relative positional relationships. The periodic and continuous nature of sine and cosine ensures that the encoding generalizes to sequences longer than those encountered during training and allows the model to infer position-dependent patterns without requiring learned parameters. This design, originally introduced in Transformer architectures, effectively captures both local and long-range dependencies in sequences. To comprehensively represent each residue within a peptide, we combined the word embedding vector with the positional encoding vector. Each component of the resulting *d_model*-dimensional vector is obtained by summing the corresponding components of the word embedding and positional encoding vectors. This fusion allows the model to capture not only the semantic relationships between amino acids but also their sequential dependencies, thereby enhancing its overall understanding of protein sequences.

### The structures of the fungi-Kcr model

**Figure 2:**
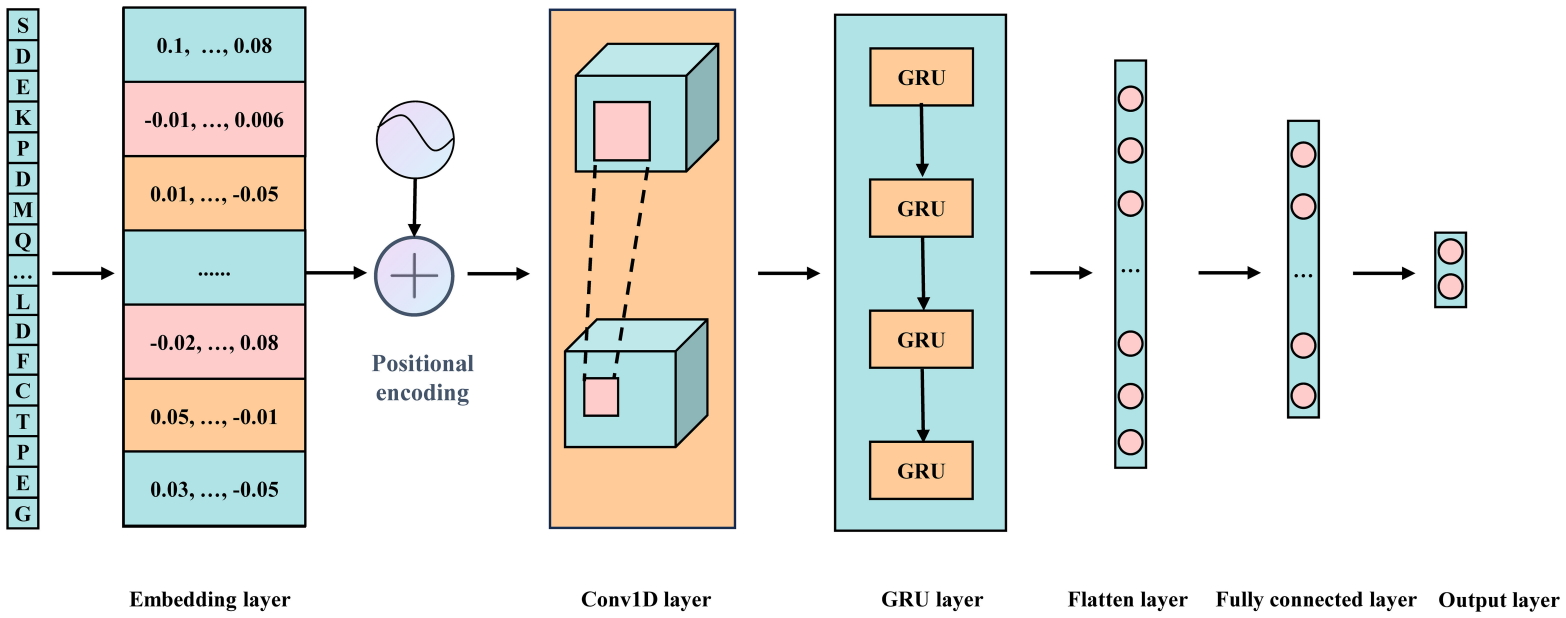
Schematic representation of the Fungi-Kcr model architecture.

The model consists of multiple modules, integrating word embedding, positional encoding, two one-dimensional convolutional layers, a GRU layer, and two fully connected layers (**Figure 2**). Additionally, various strategies are employed to effectively mitigate overfitting and ensure the model’s generalization capability. Below is a detailed explanation of each key component in the model:

Fusion of Word Embedding and Positional Encoding: The model input is a 31-dimensional integer vector. First, a word embedding layer transforms these discrete integer values into a continuous 128-dimensional vector space, forming a 31×128 embedding matrix. This transformation enriches the semantic information in the data and lays a solid foundation for subsequent processing steps. Next, the positional encoding vectors are added to each embedding vector to preserve the sequential order of elements.

One-Dimensional Convolutional Layers: To effectively extract local features from the embedding and positional encoding matrix, we employ two consecutive one-dimensional convolutional layers. The first convolutional layer maintains both input and output channel dimensions at 128, using a kernel size of 7 and a stride of 1, with ReLU activation introducing non-linearity. The second convolutional layer expands the output channel dimension to 256 while keeping the kernel size at 7 and the stride at 1, also utilizing ReLU activation. This dual-layer configuration significantly enhances the model’s ability to recognize complex local patterns.

GRU Layer: To capture long-range dependencies inherent in the sequence, the output from the convolutional layers is passed into a GRU layer. This layer has a hidden state dimension of 64, enabling it to process sequential data and generate hidden states containing contextual information. Additionally, a ReLU (Nair and GE, 2010) activation function is applied after the GRU layer to further enhance the model’s nonlinear representation capability.

Fully Connected Layers: The output from the GRU layer undergoes a flattening process, converting it into a one-dimensional vector. This vector is then processed through two fully connected layers. The first fully connected layer reduces the input dimension from 1600 (25×64) to 64 and applies a ReLU activation function. The second fully connected layer further reduces the input dimension from 64 to the final task-specific output dimension of 2, suitable for binary classification. A softmax activation function is used in the final layer to generate the probability distribution for each class.

To combat overfitting during training, multiple strategies are employed. First, Dropout layers are placed after the positional encoding, two convolutional layers, the GRU layer, and the first fully connected layer. By randomly discarding a portion of neuron outputs, the model complexity is reduced, enhancing generalization capability. The Dropout rates are carefully tuned, with a rate of 0.2 for the positional encoding layer and 0.5 for the convolutional layers, GRU layer, and first fully connected layer. Additionally, an L2 regularization term with a coefficient of 5e-4 is incorporated into the loss function to further constrain model complexity. Furthermore, batch normalization layers are introduced after each convolutional layer and the first fully connected layer to accelerate the training process and ensure model stability.

### Model optimization

We use cross-entropy as the loss function. The training process consists of 100 epochs, and the model with the lowest training loss across all epochs is selected as the final predictive model. During each epoch, the model’s weights and biases are iteratively updated using the Adam optimizer. The initial learning rate for Adam is set to 0.001 to balance convergence speed and stability.

To optimize computational resources and accelerate training, we adopt a mini-batch learning strategy, with each batch containing 128 samples. All model training and subsequent sample prediction experiments are conducted in a Python 3.10.10 environment, utilizing the PyTorch 2.0.0+cu118 deep learning framework.

### Model evaluation

The model was evaluated using 10-fold cross-validation and independent testing. For 10-fold cross-validation, the dataset was divided into 10 subsets, with each subset used once as the validation set while the remaining nine subsets were used for training. Independent testing was performed on a separate test set. Evaluation metrics included accuracy, sensitivity, specificity, F1-score, Matthews correlation coefficient (MCC), ROC curve, and area under the curve (AUC). The mathematical formulas for Sn, Sp, ACC, and MCC are as follows:


$$Sn=\frac{TP}{TP+FN}$$

$$Sp=\frac{TN}{TN+FP}$$

$$ACC=\frac{TP+FN}{TP+FN+TN+FP}$$

$$F1-Score=\frac{2\times TP}{2\times TP+FP+FN}$$

$$MCC=\frac{TP\times TN-FP\times FN}{\sqrt{\left(TP+FP\right)\times \left(TP+FN\right)\times \left(TN+FN\right)\times \left(TN+FP\right)}}$$

In the above equations, TP (true positives), FP (false positives), TN (true negatives), and FN (false negatives) represent the respective classification outcomes. Sensitivity (Sn) measures the model’s ability to correctly identify positive samples, with higher values indicating better performance in detecting positives. Specificity (Sp) evaluates the model’s ability to correctly identify negative samples, where higher values reflect improved accuracy in detecting negatives.

The F1-score provides a balanced measure of the model’s performance by considering both precision and recall, making it particularly useful in cases of imbalanced data. A higher F1-score indicates a more reliable model in distinguishing positive samples. MCC takes both sensitivity and specificity into account, with values ranging from −1 to 1. A higher MCC suggests better overall model performance.

The ROC curve graphically represents the trade-off between the true positive rate (TPR) and false positive rate (FPR) at different classification thresholds. Notably, TPR corresponds to sensitivity, while FPR is computed as 1 − specificity. The AUC quantifies the model’s ability to rank positive samples above negative ones. A higher AUC, approaching 1, indicates superior classification performance. The closer the ROC curve is to the upper-left corner, the better the model’s discriminative ability.

In this study, samples with a predicted probability greater than 0.5 are classified as positive. The evaluation metrics (Sn, Sp, ACC, F1-score, and MCC) are computed based on this fixed threshold. However, to comprehensively compare different models, we primarily rely on the ROC curve and AUC, which allow evaluation across various thresholds. The ROC curve provides an effective visualization of the balance between sensitivity and specificity, enabling direct model comparison at the same specificity levels.

To ensure the robustness of our model, we conducted rigorous testing. In ten-fold cross-validation, we computed the mean and standard deviation of each evaluation metric across folds. For the independent test, we performed 10 independent runs using different random seeds and calculated the mean and standard deviation of the results to ensure reliable and reproducible performance evaluation.

## Results

### Conservation analysis of Kcr sites in fungal species

**Figure 3:**
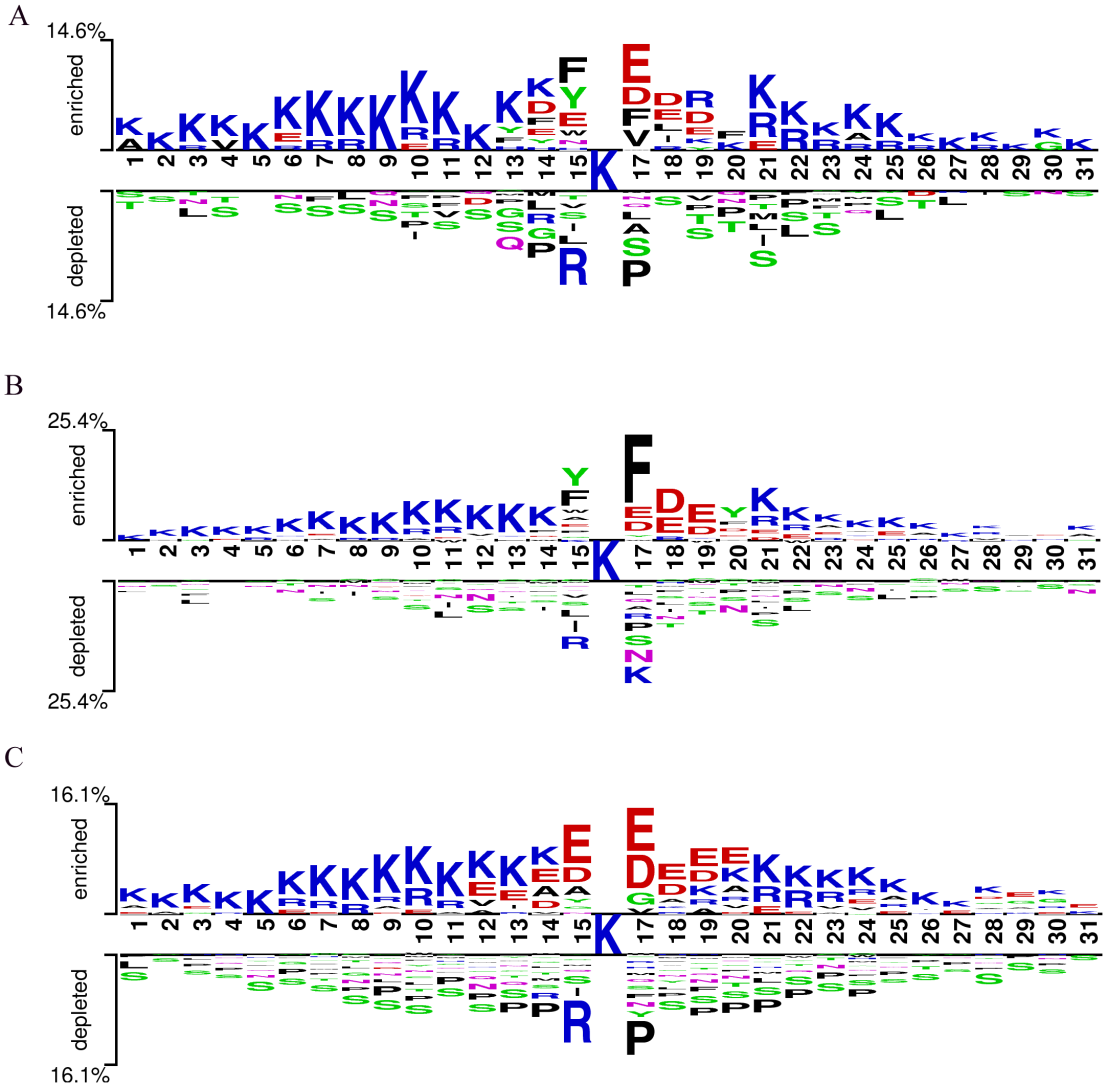
Sequence logo of Kcr sites on pathogenic fungal proteins. **(A)** Sequence logo for Botrytis cinerea; **(B)** Sequence logo for Candida albicans; **(C)** Sequence logo for Trichophyton rubrum.

The conservation of Kcr (lysine crotonylation) sites suggests their functional significance (Chen et al., 2021). Using the Two-Sample-Logo tool (Vacic et al., 2006), we analyzed the sequence motifs of Kcr sites in Botrytis cinerea, Candida albicans, and Trichophyton rubrum based on a merged training and test dataset. This dataset included 2,543 Kcr sites from Botrytis cinerea, 3,231 from Candida albicans, and 7,099 from Trichophyton rubrum, along with an equal number of non-Kcr sites selected from peptides centered on unmodified lysine residues. All peptides were standardized to 31 amino acids in length (−15 to +15 residues flanking the central lysine), ensuring consistency across species and enabling direct comparison. (**Figure 3**). Residue K was consistently overrepresented, while residue S was underrepresented across all three species. In Botrytis cinerea, residues E, D, F, and V were significantly enriched at position +1, with F, Y, and E overrepresented at position -1. Candida albicans showed a strong presence of residue F at position +1, while Y and F were the most dominant at position -1. Trichophyton rubrum displayed a prevalence of residues E and D from positions -1 to +3. Conversely, residues R and P were underrepresented at positions +1 and -1 in both Trichophyton rubrum and Botrytis cinerea, while R and K were underrepresented in Candida albicans. These findings indicate that fungal species have distinct Kcr sequence motifs compared to humans and plants, underscoring the need for a specialized prediction tool for fungal Kcr site identification.

### Performance of Fungi-Kcr on ten-fold cross-validation and independent tests

**Figure 4:**
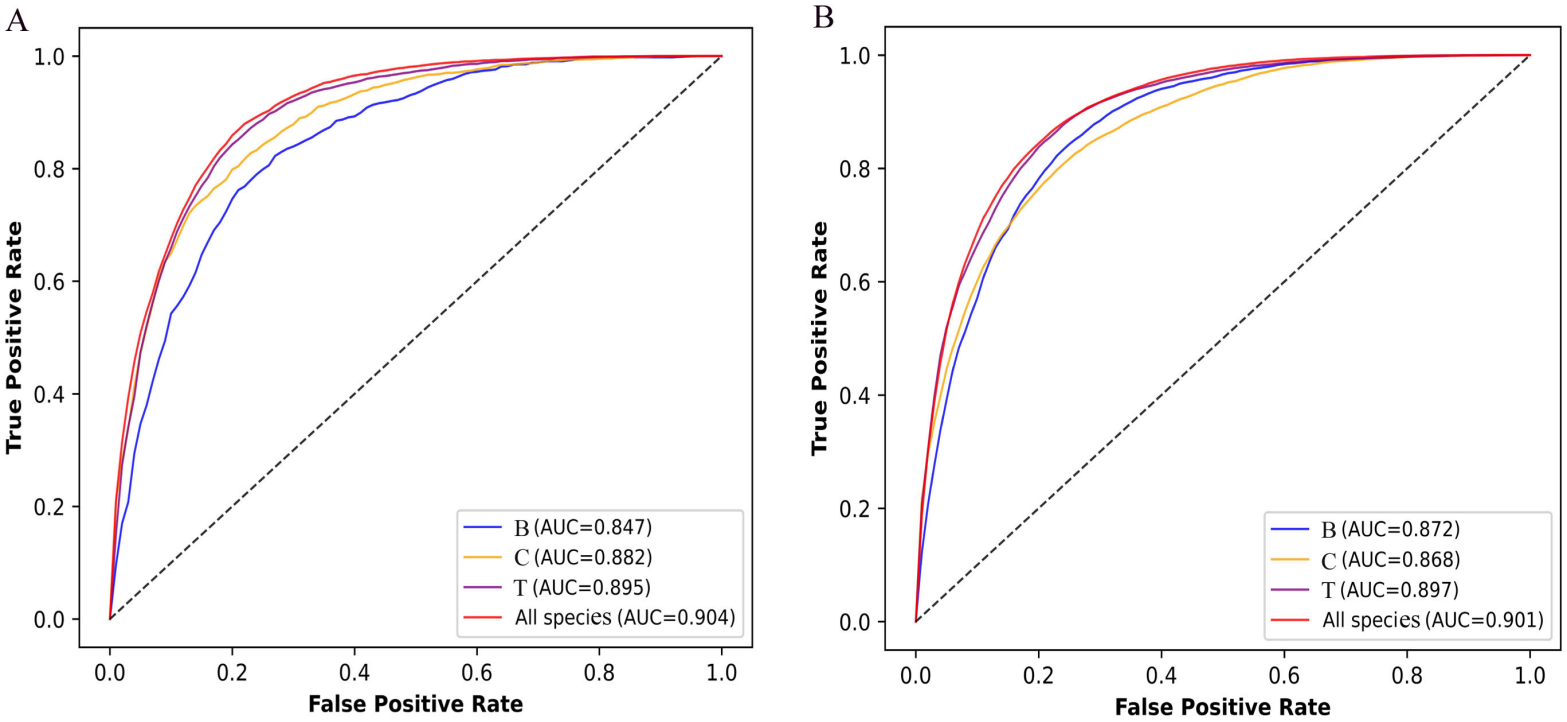
ROC curves of the Fungi-Kcr model on ten-fold cross-validation and independent tests. **(A)** The ROC curves on ten-fold cross-validation; **(B)** The ROC curve on independent tests.

To assess the performance of Fungi-Kcr, we conducted ten-fold cross-validation and ten independent tests. In cross-validation, the model achieved AUC values of 0.847, 0.882, and 0.895 for Botrytis cinerea, Candida albicans, and Trichophyton rubrum, respectively. Notably, when applied to the combined dataset, the model attained an even higher AUC of 0.904, demonstrating strong predictive capability (**Figure 4A**). The combined model exhibited robust performance, with an average sensitivity of 0.875, specificity of 0.786, accuracy of 0.830, F1-score of 0.837, and MCC of 0.663 (**Table 2**). In independent testing, the model consistently performed well, yielding AUC values of 0.872, 0.868, and 0.897 for Botrytis cinerea, Candida albicans, and Trichophyton rubrum, respectively, and an AUC of 0.901 for the combined dataset (**Figure 4B**). For the combined dataset, the model achieved a sensitivity of 0.863, specificity of 0.779, accuracy of 0.821, F1-score of 0.828, and MCC of 0.645, further confirming its robustness and reliability (**Table 3**).

**Table 2:** Metric values of the Fungi-Kcr model on ten-fold cross-validation.

Species	Sensitivity (%)	Specificity (%)	Accuracy (%)	F1-score (%)	MCC (%)	AUC (%)
Botrytis cinerea	0.774±0.029	0.768±0.041	0.772±0.017	0.773±0.014	0.544±0.036	0.847±0.019
Candida albicans	0.801±0.022	0.802±0.026	0.802±0.018	0.801±0.020	0.603±0.036	0.882±0.016
Trichophyton rubrum	0.863±0.013	0.782±0.017	0.823±0.010	0.829±0.011	0.647±0.019	0.895±0.009
Combined dataset	0.875±0.020	0.786±0.019	0.830±0.007	0.837±0.007	0.663±0.014	0.904±0.008

**Table 3:** Metric values of the Fungi-Kcr model on independent test.

Species	Sensitivity (%)	Specificity (%)	Accuracy (%)	F1-score (%)	MCC (%)	AUC (%)
Botrytis cinerea	0.760±0.021	0.815±0.016	0.788±0.003	0.781±0.007	0.577±0.006	0.872±0.002
Candida albicans	0.793±0.014	0.773±0.015	0.783±0.005	0.785±0.005	0.566±0.010	0.868±0.004
Trichophyton rubrum	0.858±0.014	0.780±0.015	0.819±0.003	0.826±0.003	0. 640±0.005	0.897±0.002
Combined dataset	0.863±0.019	0.779±0.020	0.821±0.002	0.828±0.003	0.645±0.003	0.901±0.001

Additionally, to evaluate the effectiveness of the word embedding encoding method compared to traditional feature representations, we tested the binary encoding approach using both ten-fold cross-validation and independent testing in the combined dataset. In cross-validation, the binary encoding-based model achieved an average sensitivity of 0.806, specificity of 0.800, accuracy of 0.802, F1-score of 0.803, MCC of 0.604, and AUC of 0.881. In the independent test, the performance remained consistent, with a sensitivity of 0.815, specificity of 0.793, accuracy of 0.804, F1-score of 0.806, MCC of 0.609, and AUC of 0.881.

### T-SNE visualization of Fungi-Kcr classification

**Figure 5:**
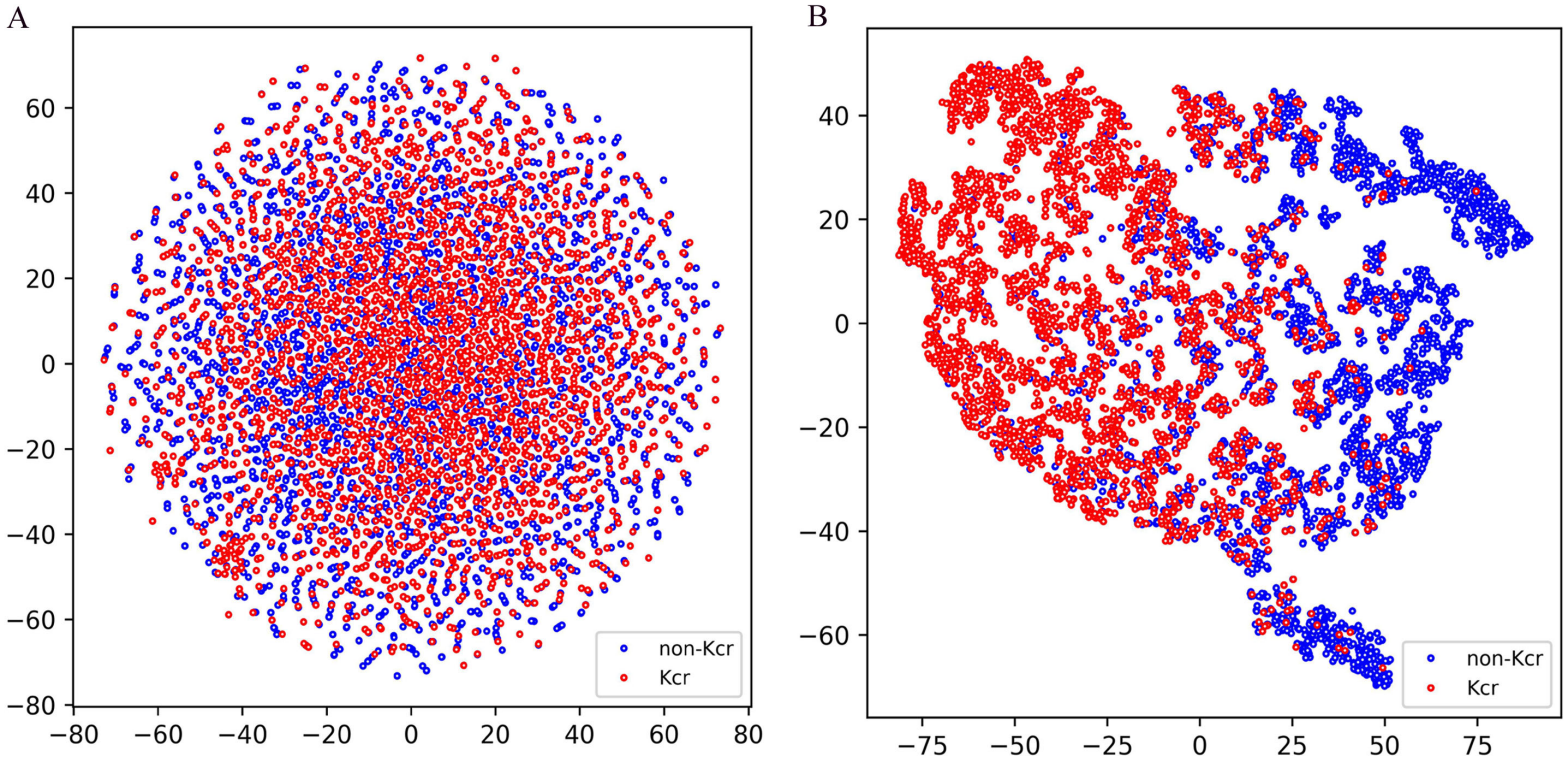
T-SNE visualization of test samples in Fungi-Kcr layers. **(A)** T-SNE visualization of input test data; **(B)** T-SNE visualization of the test set data after passing through the model’s processing modules.

To evaluate the classification performance of the model trained on the combined training set and tested on the combined test set, we employed t-distributed stochastic neighbor embedding (t-SNE) for dimensionality reduction and visualization, allowing for an intuitive understanding of the classification results. As shown in **Figure 5A**, the test set data, encoded using a binary scheme, was projected onto a two-dimensional space using t-SNE (Platzer, 2013). The distribution reveals that positive and negative samples are interwoven without a clear separation boundary, highlighting the inherent challenges of classification in the original feature space. In contrast, **Figure 5B** illustrates the distribution of the test set data after being processed through multiple layers of the model, including the embedding layer, positional encoding layer, convolutional layers, gated recurrent unit (GRU) layers, and the first fully connected layer. The processed data was then projected onto a two-dimensional space using t-SNE. Notably, a distinct separation between positive and negative samples emerged, with positive samples predominantly clustering on the left and negative samples on the right. This transformation demonstrates the model’s ability to extract meaningful representations that enhance class separability, further validating its effectiveness in distinguishing positive from negative samples.

### Comparison with conventional machine learning models

To comprehensively evaluate the performance of the Fungi-Kcr model, we compared it with traditional machine learning algorithms, including AdaBoost (Yoav and Schapire, 1997), LightGBM (Ke et al., 2017), and Random Forest (Breiman, 2001). To ensure a fair and rigorous comparison, we independently optimized hyperparameters for each baseline model using grid search combined with 10-fold cross-validation on the training dataset. For Random Forest, the hyperparameters were set to n_estimators = 500, max_depth = 10, and max_leaf_nodes = 10, with class_weight = ‘balanced’ to address class imbalance. For AdaBoost, we used n_estimators = 100. For LightGBM, the hyperparameters were n_estimators = 500, max_depth = 15, and learning_rate = 0.1, also with class_weight = ‘balanced’. The results demonstrated that Fungi-Kcr consistently outperformed all traditional methods across multiple evaluation metrics.

**Figure 6:**
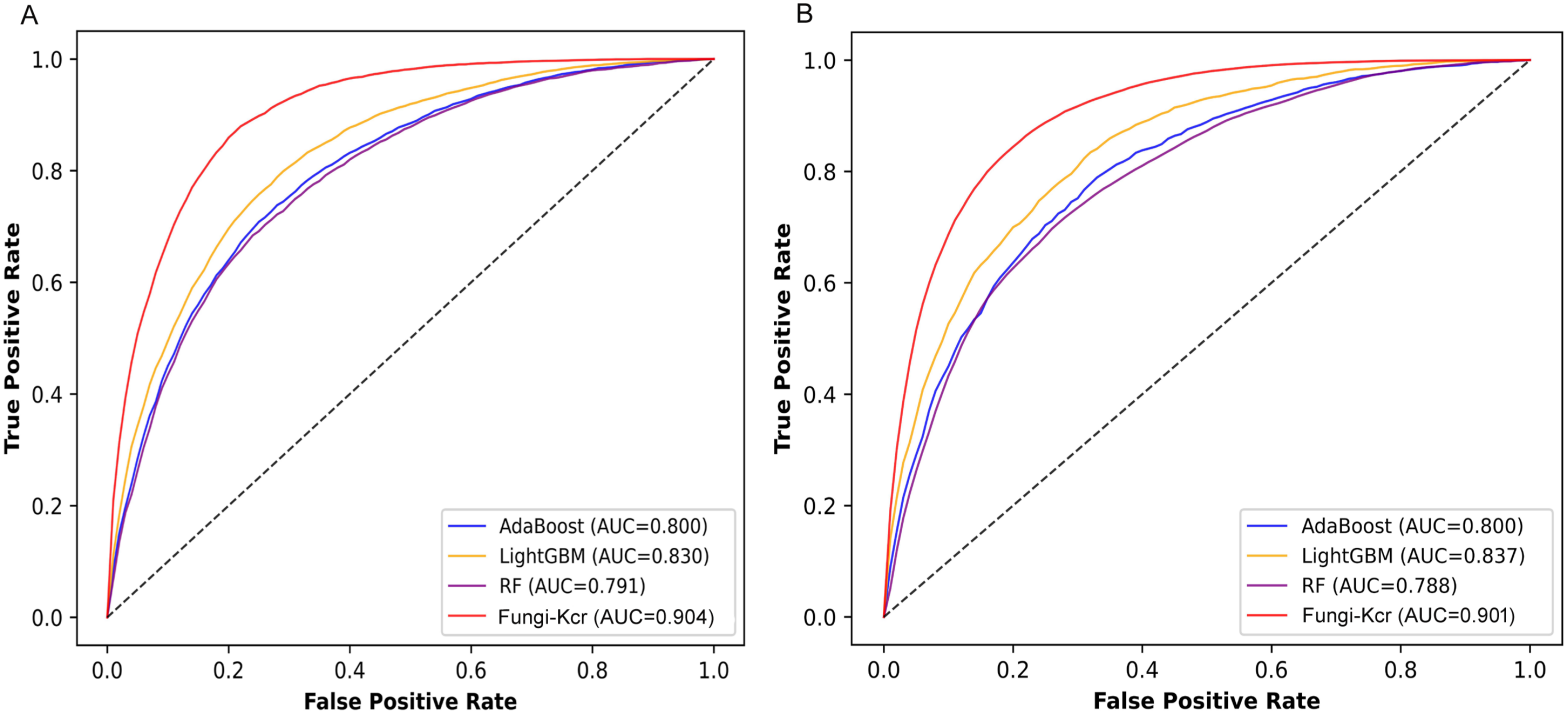
ROC curves comparing the Fungi-Kcr model with conventional machine learning methods. **(A)** ROC curves for ten-fold cross-validation; **(B)** ROC curves for independent testing.

During cross-validation (**Figure 6A**), the Fungi-Kcr model achieved the highest AUC (0.904), surpassing AdaBoost (0.800), Random Forest (0.791), and LightGBM (0.830). It also exhibited superior sensitivity, specificity, accuracy, F1-score, and MCC, highlighting its strong predictive capability (**Table 4**). Similarly, in independent testing (**Figure 6B**), Fungi-Kcr maintained its advantage, achieving an AUC of 0.901, outperforming AdaBoost (0.800), Random Forest (0.788), and LightGBM (0.837). Its consistently higher classification performance across all metrics further validates its robustness and effectiveness in distinguishing Kcr sites (**Table 5**).

**Table 4:** Comparison of Fungi-Kcr to other machine learning methods on ten-fold cross-validation.

Methods	Sensitivity (%)	Specificity (%)	Accuracy (%)	F1-score (%)	MCC (%)	AUC (%)
AdaBoost	0.708	0.752	0.730	0.724	0.460	0.800
LightGBM	0.753	0.756	0.754	0.749	0.508	0.830
Random Forest	0.684	0.760	0.722	0.711	0.445	0.791
Fungi-Kcr	0.875	0.786	0.830	0.837	0.663	0.904

**Table 5:** Comparison of Fungi-Kcr to other machine learning methods on independent test.

Methods	Sensitivity (%)	Specificity (%)	Accuracy (%)	F1-score (%)	MCC (%)	AUC (%)
AdaBoost	0.713	0.739	0.726	0.722	0.452	0.800
LightGBM	0.761	0.748	0.754	0.756	0.509	0.837
Random Forest	0.685	0.749	0.717	0.708	0.435	0.788
Fungi-Kcr	0.863	0.779	0.821	0.828	0.645	0.901

These findings underscore the superior classification capabilities of Fungi-Kcr compared to conventional machine learning methods, establishing it as a powerful tool for Kcr site prediction in fungal proteins.

## Discussion

In the present study, we developed Fungi-Kcr, the first prediction model specifically tailored for fungal proteins. Motif analysis revealed that fungal species exhibit distinct Kcr sequence motifs compared to those observed in humans and plants. Notably, each fungal species displayed its own characteristic motif pattern; however, they also shared some common features. For instance, the enrichment of acidic residues (E and D) at positions +1, +2, and +3 across all three species may increase the local negative charge, thereby promoting lysine crotonylation—a trend consistent with previous studies (Xu et al., 2017; Zheng et al., 2022). Additionally, arginine showed the lowest enrichment at position -1, possibly due to electrostatic repulsion or structural interference between the positively charged arginine and the crotonylated lysine.

The enrichment of aromatic residues (F and Y) at position -1 in Botrytis cinerea and Candida albicans might imply a role in structural stabilization or substrate recognition. Notably, previous studies have shown that certain crotonylation-recognizing proteins, such as SIRT3, can interact with the crotonyl group through π–π stacking interactions mediated by phenylalanine residues in their binding pockets (Yan et al., 2024). This suggests that the presence of aromatic residues near Kcr sites might contribute to recognition or binding by reader or eraser proteins, thereby playing a role in the regulation of this modification. The consistent depletion of proline in Botrytis cinerea and Trichophyton rubrum, known to disrupt secondary structure or alter protein conformation (Mor et al., 2016), may indicate a selective pressure against structural constraints near modification sites. Altogether, these findings highlight distinct fungal-specific Kcr motifs and suggest possible mechanistic preferences in crotonylation, emphasizing the need for a tailored prediction model.

The superior performance of the proposed model in predicting Kcr modification sites in fungal pathogenic proteins can be attributed to three key aspects: model architecture, feature processing, and regularization strategies. To better understand their contributions, we conducted an independent evaluation using the combined dataset.

The model’s deep learning architecture, consisting of an input layer, one-dimensional convolutional layers, gated recurrent unit (GRU) layers, fully connected layers, and an output layer, was designed to capture both local and global sequence features. Comparative experiments demonstrated that progressively incorporating these components significantly enhanced predictive performance. Specifically, a baseline model consisting only of an input layer, fully connected layers, and an output layer achieved an average AUC of 0.854. The addition of one-dimensional convolutional layers increased the AUC to 0.895, highlighting the effectiveness of local feature extraction. Further integration of GRU layers led to a modest improvement in AUC to 0.901, demonstrating their ability to capture long-range dependencies within the sequence. These findings underscore the critical role of model depth and complexity in improving predictive accuracy.

The model integrates both word embedding and positional encoding in its input features. While word embedding captures the semantic relationships among amino acids, positional encoding encodes the relative or absolute positional information of each residue within the sequence. Experimental results revealed that removing positional encoding led to a decrease in AUC to 0.882, suggesting that positional encoding plays a crucial role in capturing sequence structure and its influence on Kcr modification sites. This highlights its importance in enhancing the model’s ability to identify modification patterns more effectively.

To mitigate overfitting during training, the model incorporates multiple regularization techniques, including dropout, weight decay (L2 regularization), and batch normalization. Ablation studies demonstrated that omitting any of these strategies resulted in performance degradation: removing dropout reduced the AUC to 0.861, eliminating weight decay led to an AUC drop to 0.892, and excluding batch normalization similarly resulted in an AUC decline to 0.892. These findings emphasize the critical role of regularization techniques in improving the model’s generalization ability and preventing overfitting.

Even though Fungi-Kcr achieved superior performance compared to other methods, there is still room for improvement. The model currently relies solely on sequence-based features, whereas protein structural information has been shown to enhance post-translational modification site prediction (Mattei et al., 2001; Liang et al., 2023; Ertelt et al., 2024; Qin et al., 2024). Incorporating protein 3D structural features or contact maps could further improve the model’s performance. For instance, predicted secondary structure (e.g., via PSIPRED (McGuffin et al., 2000)), solvent accessibility (e.g., from ASAquick (Faraggi et al., 2014) or NetSurfP-3.0 (Hoie et al., 2022)) or evolutionary profiles (e.g., PSSM (Zhan et al., 2020)) could help enrich the feature space. Furthermore, incorporating protein-protein interaction network features—such as degree centrality or functional modules—may reveal whether Kcr preferentially occurs on network hubs or in specific pathways. In future work, these structural and network-based features could be encoded alongside sequence features using multimodal learning strategies, such as feature-level concatenation or attention-based architectures.

Although this study focused on three fungal species with extensive experimentally verified Kcr data, we acknowledge that this taxonomic scope may limit the generalizability of the findings. Expanding the analysis to include additional fungal species—especially those from diverse evolutionary lineages—could reveal further conserved or lineage-specific Kcr patterns. Moreover, cross-species comparisons may enhance our understanding of the evolutionary constraints on Kcr motifs and test the robustness of the Fungi-Kcr model across different levels of sequence homology. We intend to pursue these directions in future research to further validate and refine the predictive utility of our model.

To further understand the limitations and areas for improvement of the Fungi-Kcr model, future work will include an in-depth analysis of misclassified samples, including both false positives and false negatives. This investigation may help identify specific sequence patterns or contextual features contributing to incorrect predictions. For instance, false positives could result from unannotated or conditionally modified lysines, while false negatives might reflect limitations in the sequence window size or insufficient representation of rare motifs in the training data. A deeper examination of these cases would offer valuable insights into performance bottlenecks and inform future enhancements in feature engineering or model architecture.

Additionally, the model does not explicitly distinguish between histone and non-histone Kcr sites, despite evidence suggesting different modification patterns between these protein types (Chen et al., 2021; Khanal et al., 2023; Jiang et al., 2024). Developing separate models for histone-specific and non-histone-specific Kcr modifications may lead to better predictive accuracy. Another limitation is that the dataset may contain an imbalanced distribution of Kcr sites among different bacterial species, which could introduce species-specific biases. Expanding the dataset to include a broader range of bacterial species and ensuring a balanced sample distribution could improve robustness. The generalizability of the model to other organisms is also uncertain, as it is specifically designed for fungal proteins. Future research should explore whether the model can be adapted to other species, such as plants and mammals. Integrating multi-omics data, such as proteomics, epigenomics, and transcriptomics, may provide additional contextual information for more accurate predictions. Furthermore, the development of a user-friendly web server or standalone software would allow researchers to use the model for large-scale Kcr site predictions without requiring extensive computational resources. While Fungi-Kcr represents a significant advancement in Kcr site prediction, addressing these limitations and incorporating future improvements will be crucial for further enhancing its predictive capabilities and expanding its applications in bacterial protein research.

## Conclusion

In this study, we developed Fungi-Kcr, a deep learning-based model for predicting lysine crotonylation sites in pathogenic fungi. The model integrates convolutional neural networks, gated recurrent units, and word embedding, leveraging biologically relevant sequence features to enhance predictive accuracy. Our results demonstrate that Fungi-Kcr outperforms conventional machine learning methods and species-specific models, achieving robust performance across diverse fungal species. Importantly, this study provides a valuable tool for understanding crotonylation in fungal pathogens, which may offer new insights into their regulatory mechanisms. As more experimentally validated Kcr sites become available and deep learning techniques continue to advance, further refinements of Fungi-Kcr are expected to improve its predictive power and biological applicability.

## Data Availability

The original contributions presented in the study are included in the article/**Supplementary Material**. Further inquiries can be directed to the corresponding authors.
