# Supplementary figures and images for: Fungi-Kcr: a language model for predicting lysine crotonylation in pathogenic fungal proteins
Source: Front Cell Infect Microbiol. 2025 Jul 15;15:1615443. doi: 10.3389/fcimb.2025.1615443 (PMC12303977; doi:10.3389/fcimb.2025.1615443)

**AUC vs Window Length (Smooth Curve)**

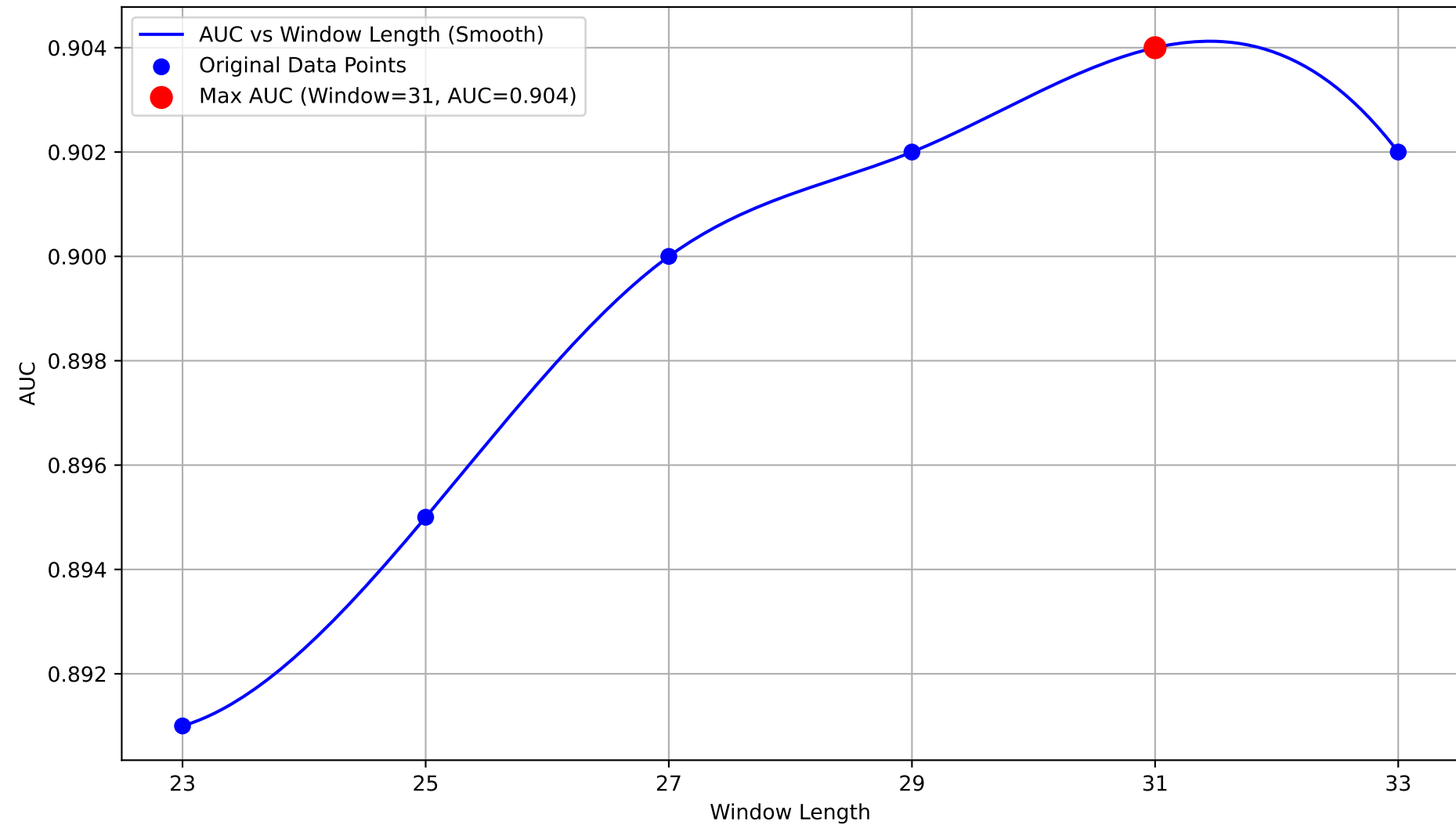

Supplement: Supplementary file 1 [file DataSheet1.pdf]
